# Ubiquitin Signaling: Extreme Conservation as a Source of Diversity

**DOI:** 10.3390/cells3030690

**Published:** 2014-07-10

**Authors:** Alice Zuin, Marta Isasa, Bernat Crosas

**Affiliations:** 1 Institut de Biologia Molecular de Barcelona, CSIC, Barcelona Science Park, Baldiri i Reixac 15-21, 08028 Barcelona, Spain; E-Mail: alice.zuin@ibmb.csic.es; 2 Department of Cell Biology, Harvard Medical School, Longwood, Boston, MA 02115, USA; E-Mail: isasa@hms.harvard.edu

**Keywords:** ubiquitin, proteasome, protein degradation, evolution, eukaryotes

## Abstract

Around 2 × 10^3^–2.5 × 10^3^ million years ago, a unicellular organism with radically novel features, ancestor of all eukaryotes, dwelt the earth. This organism, commonly referred as the *last eukaryotic common ancestor*, contained in its proteome the same functionally capable ubiquitin molecule that all eukaryotic species contain today. The fact that ubiquitin protein has virtually not changed during all eukaryotic evolution contrasts with the high expansion of the ubiquitin system, constituted by hundreds of enzymes, ubiquitin-interacting proteins, protein complexes, and cofactors. Interestingly, the simplest genetic arrangement encoding a fully-equipped ubiquitin signaling system is constituted by five genes organized in an operon-like cluster, and is found in *archaea*. How did ubiquitin achieve the status of central element in eukaryotic physiology? We analyze here the features of the ubiquitin molecule and the network that it conforms, and propose notions to explain the complexity of the ubiquitin signaling system in eukaryotic cells.

## Abbreviations

Ubiquitin proteasome system (UPS); Small Archaeal Modifier Proteins (SAMPs); Isoleucine (Ile); Aspartic acid (Asp); Histidine (His); Valine (Val); Deubiquitinating (DUB) enzyme; Open reading frames (ORFs); Ubiquitin-activating enzyme (E1); Ubiquitin-conjugating enzyme (E2) ; Ubiquitin-ligating enzymes (E3); Ubiquitin interacting motif (UIM); Inverted UIM (IUIM or MIU); Ubiquitin-binding zinc (UBZ) finger; Ubiquitin-associated (UBA) domain; Coupling of ubiquitin conjugation to the endoplasmic reticulum degradation (CUE) domain; Ubiquitin conjugating (UBC) enzyme; Ubiquitin E2 variants (UEV); GRAM-like ubiquitin-binding in EAP 45 (GLUE) domain; Pleckstrin homology (PH) domain; Plekstrin homology receptor for ubiquitin (PRU); Homologous to the E6-AP Carboxyl Terminus (HECT); Really Interesting New Gene (RING); RAB5 guanine nucleotide exchange (RABEX5) factor; Residual dipolar couplings (RDCs); Ubiquitin C-terminal hydrolases (UCHs); Ubiquitin-specific proteases (USPs); Ovarian tumor (OTU) protease; Machado-Josephin domain (MJD) protease

## 1. Extreme Conservation of Ubiquitin

Ubiquitin is a post-translational protein modifier described during the 1980s as a cofactor of a proteolytic system, today known as the Ubiquitin Proteasome System (UPS) [1]. Evidence of the involvement of ubiquitin in cell functions has increased in the last decades and now it is widely accepted that ubiquitin is one of the central elements of eukaryotic cell physiology. Ubiquitin regulates multiple pivotal cellular processes, such as proteasomal-dependent protein degradation, endocytosis, autophagy, cell cycle, DNA stability, traffic, metabolic pathways, transcription, and translation [2]. The causes of the high conservation between ubiquitin gene products among distant eukaryotic species, as compared to other conserved proteins, are still unclear. As discussed below, most likely, the properties of ubiquitin were positively selected and fixed in early stages of evolution of an eukaryotic ancestor [3]. Moreover, the structure of ubiquitin genes, organized in tandems and in multiple loci, facilitated a strong concerted evolution [4,5].

The ubiquitin signaling system is mediated by an enzymatic cascade that modifies substrate proteins with ubiquitin, process known as ubiquitination. This process is catalysed by the sequential activity of ubiquitin-activating (E1), ubiquitin-conjugating (E2), and ubiquitin-ligating (E3) enzymes. Protein ubiquitination generates a complex signaling code in modified proteins, which includes the formation of distinct types of ubiquitin polymers, covalently conjugated to the substrate (polyubiquitination), or the formation of ubiquitin-protein conjugates with one single ubiquitin molecule (monoubiquitination). Ubiquitinated substrates will be subsequently targeted into distinct pathways depending on the type of ubiquitin tag they carry [2,6].

**Figure 1:**
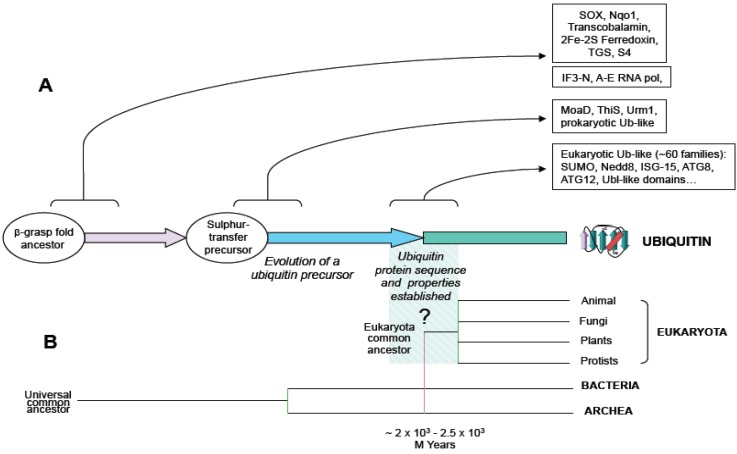
Schematic representation of ubiquitin evolution from an ancestral beta-grasp fold gene. (**A**) Beta-grasp fold is found in multiple protein families in prokaryotes and eukaryotes. Arrows show functional diversification at the levels of: beta-grasp fold ancestor, sulphur transfer precursor and ubiquitin-type families, and boxes contain groups of proteins, as reported in (3). (**B**) An evolutionary tree including branches of main groups is shown, and the origin of eukaryotes is hypothetically linked to the origin of ubiquitin.

For many years the paradigm that prevailed was that ubiquitin signaling system was only found in eukaryotes. However, recent works showing that bacteria and archaea contain ubiquitin and ubiquitin-like systems with the capacity to generate protein conjugates draw a new map of ubiquitin signaling evolution. First, it was shown that the existence of prokaryotic sulphur-carrier proteins, MoaD and ThiS, were involved in molybdenum cofactor and thiamin biosynthesis, respectively. MoaD and ThiS are structurally related to ubiquitin, sharing a beta-grasp folding and a glycine-glycine C-terminal end, and activated by a mechanism similar to ubiquitin activation (Figure 1) [3,7,8,9]. Nonetheless, in MoaD and ThiS pathways, sulphur-containing factors are the final product, whereas, in eukaryotic ubiquitin and ubiquitin-like systems, sulphur-conjugation is an intermediary step in the formation of a final suplhur-free protein-protein conjugate.

Later, the description of SAMPs, ubiquitin-like protein modifiers, in the archaea *Haloferax volcanii*, showed unequivocally that protein conjugation by ubiquitin-like proteins is not exclusive to eukaryotes [10]. SAMP conjugation does not appear to require E2 and E3 conjugating factors, being the E1 enzyme competent for sampylation. Interestingly, mutants showing decreased proteasome activity in *H. volcanii* accumulate SAMP conjugates, indicating that SAMPs may play a role in proteasome-dependent degradation [10]. *H. volcanii*, and most archaea species, encode only one E1-like protein. This E1-like factor has to act, at least in *H. volcanii*, as a multifunctional activator for distinct ubiquitin-like proteins, playing roles in sampylation and in sulphur mobilization. Thus, a very interesting scenario from the evolutionary and mechanistic points of view is defined, in which ubiquitin-like signaling systems are at the crossroads between sulphur mobilization and protein conjugation. Although a multifunctional system was first found in *H. volcanii*, it could exist in other archaea species and it shows analogies with the Urm1 system of protein conjugation and sulphur transfer in eukaryotes [11,12]. The yeast protein Urm1, similar to ThiS and MoaD, acts both as a sulphur carrier in a tRNA modification pathway and as a protein modifier, and is considered a possible evolutionary link between prokaryotic sulphur carrier and eukaryotic ubiquitin-like protein [11,13].

**Figure 2:**
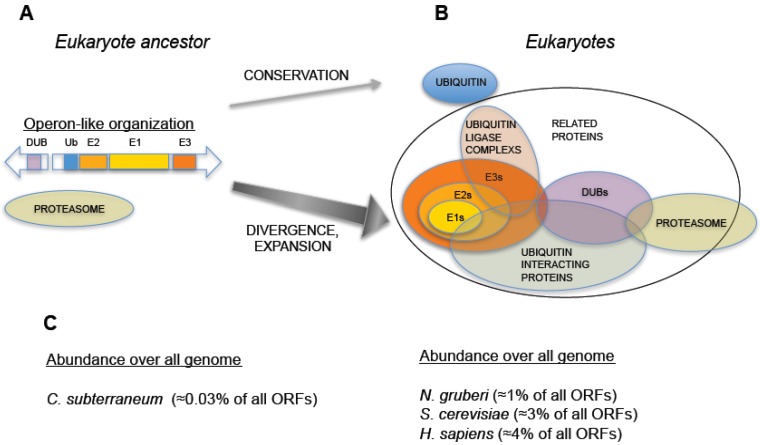
Hypothetical evolution of the Ubiquitin-Proteasome System from an operon-like cluster. (**A**) Schematic representation of an operon-like cluster, based on *C. subterraneum* (11), in which the minimal representation of the factors required for ubiquitin-signaling is found in this type of genetic arrangement. (**B**) Representation of an expanded UPS, as found in eukaryotes, which includes groups of factors involved in UPS and their interactions. Central arrows indicate distinct behaviors in UPS evolution: while ubiquitin was conserved, the rest of ubiquitin signaling genes underwent an expansive process. (**C**) Percentage of ubiquitin and UPS related genes (including ubiquitin-like related factors) with respect to all annotated open reading frames in the genomes of: *C. subterraneum* (as a hypothetical model of eukaryote ancestor), the protist *N. gruberi*, the yeast *S. cerevisiae* and *H. sapiens*. A dramatic increase in the abundance of UPS genes is observed in eukaryotes.

An additional striking discovery concerns another archaeal species, *Caldiarchaeum subterraneum.* The genome of *C. subterraneum* was sequenced and the existence of a full set of ubiquitin signaling factors was found: one single-copy ubiquitin gene, one ubiquitin activating enzyme (E1), one ubiquitin conjugating enzyme (E2), one RING-type ubiquitin-protein ligase (E3), and one deubiquitinating enzyme related to the proteasome subunit Rpn11 (described below) [14]. Interestingly, these five genes are organized in an operon-like cluster, representing the most simplified genetic arrangement encoding a eukaryote-like ubiquitin signaling system (Figure 2) [14]. Several phylo-genetically diverse bacteria (of the phyla Actinobacteria, Planctomycetes, and Acidobacteria) also carry related operons [15]. Overall, these uncharacterized ubiquitylation operons are sporadically dispersed in bacteria and archaea, and are often missing in close relatives. Further research will be required to determine whether the operon-like topology is a good model for an ancestral pre-eukaryotic UPS [16]. To date, it is the strongest evidence of a functionally organized UPS in a non-eukaryotic species.

It is worth noting that the operon organization found in archaea is not found in eukaryotic species, which always show a higher number and a spread out distribution of ubiquitin signaling system genes. In eukaryotes, ubiquitin is redundantly expressed in genomes, encoded as a fused protein by at least three loci: a polymeric head-to-tail concatamer of multiple (approximately from 4 to 15) ubiquitin open reading frames and two fusions with L40 and S27 ribosomal proteins [4,17,18]. The enzymes involved in ubiquitin signaling also define consistent E1 < E2 < E3 pyramidal networks, and are widely distributed in eukaryotic genomes. For example, the amoebo-flagellate *Naegleria gruberi*, that belongs to the protist clade heterolobosea, emerged from other eukaryotic lineages over 10^3^ million years ago, contains more than 100 ubiquitin signaling system genes [19], including multiple E2s and E3s, and most of ubiquitin related enzymatic families and factors found in higher eukaryotes. The same can be concluded when analyzing the composition of genomes from other protists, fungi, plants, and animals. Therefore, in an ancestral pre-eukaryotic cell, a single-copy ubiquitin gene and ubiquitin signaling genes, perhaps from an operon-like cluster, underwent several duplication and recombination events that generated redundancy in ubiquitin genes and the multiplicity of ubiquitin signaling genes (Figure 2).

## 2. Which are the Functional Features of Ubiquitin that Eukaryotes have Preserved so Strictly?

**Figure 3:**
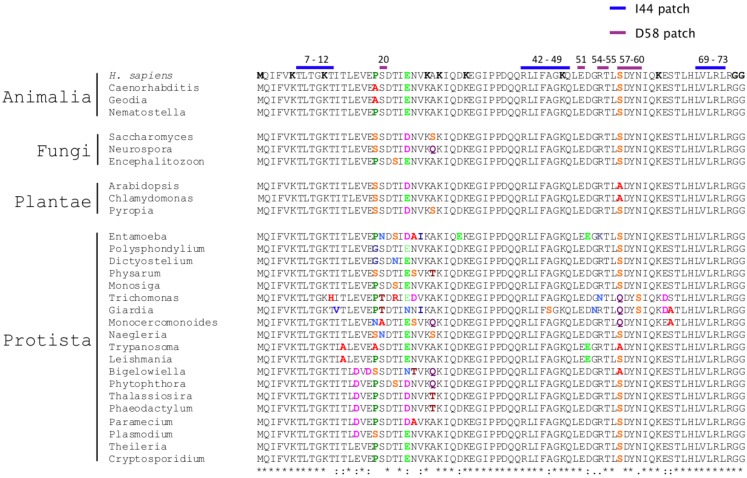
Strict conservation of functional traits of ubiquitin through evolution. Multiple alignment of eukaryotic ubiquitin protein sequences is shown. Proteins are grouped by eukaryotic kingdoms. On the top of the alignment, amino acids composing ubiquitin interactive surfaces (isoleucine 44 and aspartate 58 patches) are shown in blue and violet, respectively. The first aligned sequence, from H. sapiens, contains in bold the N-terminal methionine, the seven lysine residues and the glycine-glycine C-terminal motif involved in ubiquitin conjugation. At the bottom, Clustal similarity symbols are included. Within the alignment, positions that show no variation are shown in black. Amino acid variations are highlighted in colors.

Despite ubiquitin gene redundancy, no drift is observed, and eukaryotic genomes keep identical copies of the ubiquitin gene. The coexistence of nearly identical redundant ubiquitin coding sequences within eukaryotic species fits in a model of strong concerted evolution [4,5]. Concerted evolution prevents, by homologous recombination, the incorporation of mutations in highly conserved genes, which show redundant copies in genomes. This mechanism blocks the divergence of paralog genes, which show higher conservation than with the ortholog genes from different species. Ubiquitin genes fit this model with the caveat of the ubiquitin-S27 fusion, which shows a pattern of divergence in several groups [18]. Comparison of genomes from different eukaryotic groups also shows extreme low divergence in ubiquitin protein sequence, even when highly distant species are compared, and shows virtually no variations when sequences within ‘crown’ groups are compared (Figure 3). Therefore, ubiquitin did not acquire major novel features during eukaryote radiation. *Ergo*, ubiquitin characteristics were likely selected in a previous period.

Structurally, ubiquitin belongs to the superfamily of beta-grasp folded proteins, consistent in four or five beta strands forming an anti-parallel sheet and one alpha helix region (Figure 1). A common feature of the beta-grasp fold is the adjacent and parallel orientation of the N- and C-terminally located strands, crossed by the helical group, defining the center of the molecule. This structural pattern of ubiquitin is stabilized by hydrophobic interactions (conserved in all the members of the beta-grasp folded proteins), providing a compact architecture, highly resistant to proteolytic processing, stable to temperature and pH changes [20,21].

From the functional point of view, eukaryiotic ubiquitin mainly preserves two basic features: non-covalent recognition of surfaces and covalent attachment to a protein substrate. Remarkably, multiple protein domains have converged in evolution to bind the same regions of ubiquitin, and different enzymatic families have evolved to accommodate ubiquitin surfaces in their active sites in order to catalyze ubiquitin conjugation or deconjugation reactions [9,22]. Ubiquitin conjugation is achieved by means of a protein-protein ligation reaction, in which the donor is the C-terminal end of ubiquitin, which shows a conserved glycine-glycine motif, and the acceptor is generally the amino group of a lysine residue of the protein substrate. The covalent bond established in the reaction of ubiquitination, described below, is an iso-peptide bond. The combination of recognition of surfaces by non-covalent interaction and protein modification define a network in which ubiquitin is the central node, and indeed, the full ubiquitin signaling system is based on that.

## 3. Ubiquitin-Interacting Universe

The nature of ubiquitin recognition is divers and very complex, including distinct well-characterized interactive regions in ubiquitin surface. Remarkably, one ubiquitin region, the isoleucine 44 (Ile44) patch (Figure 3), centralizes most of the interactions with ubiquitin binding domains [22]. This surface binds more than a dozen of structurally and evolutionarily distinct types of ubiquitin-binding domains, distributed in hundreds of coding sequences in eukaryotic genomes, and involved in multiple cellular processes, such as DNA stability, endocytosis, transcriptional control, proteasome regulation, cell cycle progression, to give a short list [22,23]. This evidence illustrates the strong commitment of a single ubiquitin surface to eukaryotic cell physiology. Interestingly, domains binding the Ile44 patch have reached this condition by convergent evolution and they are found in distinct structural contexts. For example, three ubiquitin-binding domains, the ubiquitin interacting motif (UIM), the inverted UIM (IUIM or MIU), and the ubiquitin-binding zinc finger (UBZ) use one single alpha-helix to accommodate ubiquitin [24,25,26]. The ubiquitin-associated domain (UBA) and the coupling of ubiquitin conjugation to the endoplasmic reticulum degradation domain (CUE) bind ubiquitin by means of two discontinuous alpha-helices [27,28]. Ubiquitin conjugating enzymes (UBCs) and UBC variants, such as the ubiquitin E2 variants (UEV), use beta-sheets, and the GRAM-like ubiquitin-binding in EAP45 domain (GLUE) interacts with ubiquitin using residues from multiple structural motifs: one PH domain, two beta-strands, one alpha-helix and a loop [29]. The plekstrin homology receptor for ubiquitin (PRU) found in Rpn13 proteasomal subunit also utilizes a PH domain and three loops to interact with ubiquitin [30]. The ubiquitin binding region of NEDD4 HECT ubiquitin ligase interacts with ubiquitin by means of a tripod involving three distinct loops [23,31]. Two additional surfaces of ubiquitin, the Asp58-centered region [25,32], and the C-terminal surface [33], recognize additional ubiquitin binding domains, such as the ZnF domain of RAB5 guanine nucleotide exchange factor (RABEX5) and the ZnF domain of Isopeptidase T, respectively.

Which is the basis for the high interactive capacity of ubiquitin surfaces? Combined structural and dynamics studies suggest that ubiquitin surface exhibits a fine balance between rigidity and plasticity [34,35]. Structural ensemble of multiple ubiquitin structures, refined against residual dipolar couplings (RDCs), support a model in which molecular recognition dynamics of ubiquitin is based on conformational selection instead of induced fit [34]. Thus, the capacity of ubiquitin to bind partners with high affinity and specificity could be explained by flexible surfaces centered around rigid hotspots [34]. Mobility of flexible side-chains with a liquid-like behavior of both exposed and unexposed amino acid residues, suggest also a high capacity to contact partners [35]. Lange and collaborators found that Ile44 and the neighbor His68 are rigid, whereas the rest of side-chains within the interacting region, such as Leu8 and Val70, show high flexibility. Moreover, the Asp58 binding region also appears to be as rigid as Ile44. Therefore, ubiquitin structure has integrated folding and surface properties to optimize interface definition. This interactive capacity of ubiquitin could be one of the pillars of the asymmetric complexity of the UPS, which built a highly extended interactome relying on a single molecule.

## 4. Diversity of Ubiquitin-Based Enzymology

Ubiquitin also conserves all features required for covalent auto-modification or modification of other proteins: the C-terminal tail motif, formed by the glycine-glycine doublet, and the chain forming residues, namely, the N-terminal methionine and lysines at positions 6, 11, 27, 29, 33, 48, and 63 [36], which are present in all eukaryotes (Figure 3). When conjugated, ubiquitin generates a complex input of functional information to modified proteins, which has been defined by some authors as the ubiquitin ‘code’ [22,37]. Conjugation of one single ubiquitin moiety, known as monoubiquitination, triggers protein interactions, localization and modulates protein activity, becoming a mechanism of signal transduction. Polyubiquitination in Lys48 and Lys11 targets proteins to the proteasome. N-terminal and Lys63 polyubiquitination is involved in lysosome targeting, DNA repair and activation of the Nf-kB signaling pathway. Finally, polyubiquitination in lysines 6, 27, 29, and 33 is not so well understood, with putative roles in protein degradation and DNA repair.

The process of modification involves an enzymatic cascade that includes E1, E2, and E3 enzymes. Again, these enzymes belong to structurally and evolutionarily distinct protein families that share the ability to promote ubiquitin-based enzymology. E1 enzymes belong to the superfamily of ubiquitin-like activating enzymes, which adopt a Rossmann fold [38]. Initially, C-terminal glycine ubiquitin is activated by E1, forming a ubiquitin-AMP adduct that remains bound to the enzyme. The active site cysteine residue of the E1 attacks the C-terminus of ubiquitin, producing a thioester intermediate, which attaches ubiquitin to E1. Activated ubiquitin is transferred to the cysteine active site of an E2, preserving the thioester bond and mediating the interaction with the E3, which promotes the formation of an isopeptide bond between ubiquitin C-terminus and the epsilon-amino group of a lysine residue of the modified protein. Canonically, E3 factors recognize protein substrates by binding to specific motifs or surfaces of the protein substrate. Two types of E3 enzyme approach ubiquitin ligation differently. HECT ligases contain a cysteine active site similar to the one of E2 and, during catalysis, activated ubiquitin moieties are transferred to their E3 active sites. Alternatively, RING ligases adapt E2-ubiquitin intermediates, which will ultimately promote ligation.

Ubiquitination constitutes a chemically stable protein modification, which may be only removed by the action of proteases specialized in isopeptide processing. These isopeptidases or deubiquitinating enzymes (DUBs) perform a nucleophylic attack to the carbonyl group of the isopeptide bond. Two types of DUBs have been described: cysteine and zinc active site enzymes. DUBs with cystein active contain a catalytic triad, in which an aspartic acid polarizes a histidine residue, which deprotonates the cystein. This mechanism is found in four families of DUBs: ubiquitin C-terminal hydrolases (UCHs), ubiquitin-specific proteases (USPs), ovarian tumor proteases (OTUs) and Josephins (MJDs). In zinc active sites of DUBs, the zinc atom is attached to two histidine residues and one aspartate residue, and one polarized water molecule coordinates the forth link to the metallic atom, similarly to other metalloenzymes. These types of active sites are found in JAB1/MPN/MOV34 (JAMMs) DUB enzymes. Interestingly, the simplest ubiquitin system found in archaea contains a representative of the JAMM family, a form with high homology with the proteasomal DUB Rpn11 [14], involved in protein degradation and ubiquitin recycling [39,40].

Diversification of ubiquitin-based enzymatic families in eukaryote evolution certifies the complexity of the system. The most striking expansion is found in ubiquitin-protein ligases, the largest enzymatic group within the UPS. For example, the human genome contains more than 600 RING-ligase family related genes and 28 HECT-ligase family genes, while less than 100 DUBs [41,42,43]. If the archaeal operon organization is used as a reference, the RING would be the oldest gene family. Interestingly, RING ubiquitin-ligases do not contain an enzymatic active site. RING E3 factors coordinate the interaction of an E2~ubiquitin enzyme and the protein substrate, by placing them in proximity and in a productive manner. Based on the number of representatives, RING ligases have been very successful, and, indeed, RING domains are part of monomeric, dimeric and multimeric factors that promote ubiquitin-protein ligation in hundreds of substrates. On the other hand, HECT ligases, which contain a cysteine active site, would be acquired later by early eukaryotes. The protist N. gruberi has 12 HECT annotated genes, and animals and plants contain multiple non-monophyletic HECT gene groups [44,45], showing the wide distribution of these types of enzyme, as well.

## 5. Concluding Remarks

**Figure 4:**
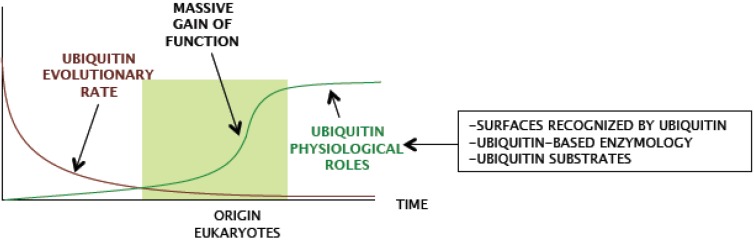
Hypothesis of a massive gain of function of ubiquitin in early eukaryotes. A schematic representation of the hypothetical behavior of ubiquitin evolutionary rate and ubiquitin physiological roles during time is shown. Ubiquitin evolved from ancestral forms of beta-grasp folded proteins (see also Figure 1), and its evolutionary rate decreased to a minimum, coincident with the origin of eukaryotes. Concomitantly, ubiquitin physiological roles increased massively, which resulted in a dramatic increase of ubiquitin signaling related genes in eukaryotic genomes.

The adaptations that involved the origin of the eukaryotic life are main aspects of biology still under debate. The ubiquitin and ubiquitin-like signaling systems are a complex interactive and enzymatic network, representing approximately from 1% (*N. gruberi*, and other protists) to 4% (*H. Sapiens*) of all ORFs annotated in genomes, that relies on ubiquitin sequence stability (Figure 2). This represents a tremendous level of negative selection on ubiquitin genes, and this selection is acting on ubiquitin since the origin of eukaryotes. Remarkably, ubiquitin, which has virtually not changed in more than 2 × 10^3^ million years, has performed during this period distinct biological functions in heterogeneous and changing cellular environments. A likely scenario is that a massive gain-of-function of ubiquitin-dependent processes in early steps of eukaryote ancestor evolution caused the commitment of ubiquitin in multiple essential cellular roles and the extreme conservation of ubiquitin sequence along eukaryotic evolution (Figure 4). In parallel, ubiquitin-based enzymology, ubiquitin-interacting surfaces, and in general, ubiquitin-related proteins underwent a marked expansion. Therefore, even though ubiquitin has been an immobile factor of eukaryotic system, it has become a source of molecular diversity and elaborated signaling. This *anisotropic* evolution has had huge impact in eukaryotic physiology, since ubiquitin, far from being confined to a reduced number of functions, is imbedded in almost all cellular processes in eukaryotes.
